# Semen quality among men attending urology services in the Dschang Health District, west Cameroon: A retrospective study on 379 cases

**DOI:** 10.18502/ijrm.v18i2.6419

**Published:** 2020-02-27

**Authors:** Aimé Césaire Momo Tetsatsi, Désiré Alumeti Munyali, Georges Romeo Bonsou Fozin, Esther Ngadjui, Modeste Wankeu-Nya, Pierre Watcho

**Affiliations:** ^1^Animal Physiology and Phytopharmacology Laboratory, University of Dschang-Cameroon, Dschang.; ^2^Department of Animal Organisms Biology, University of Douala-Cameroon, Dschang.

**Keywords:** Sperm damage, Male infertility, Semen analysis, Dschang Health District.

## Abstract

**Background:**

Infertility is a common condition affecting at least 15% of couples worldwide, and male factors are involved in about half of this prevalence rate. In Cameroon, about 20%-40% of couples are the victims. However, the sperm characteristics of infertile men are yet to be described in the health districts in Cameroon for better management of male infertility.

**Objective:**

The present study was designed to assess the sperm profile and related sociodemographic factors of men attending the urology services at the Dschang Health District.

**Materials and Methods:**

It consisted of a 10 yr retrospective study carried out in the Dschang Health District. The results of patients' semen analysis (SA) were computed using Epi Info software and expressed as qualitative and quantitative spermogram state as described by the clinician and sociodemographic features of those patients.

**Results:**

Out of the 379 patients studied, 83.91% had abnormal spermogram. Patients older than 50 yr were the most affected when grouped into age categories. With regard to patient's profession, 52.51% had specified their profession and from that group, although farmers (9.31%) represented the lowest size category, they were the most affected with 94.74% having abnormal spermogram.

**Conclusion:**

This study indicates that the sperm damage is the major cause of male infertility in the Dschang Health District. It also shows that farmers are the most affected category and it could be linked to the long-term exposure to pesticides. These results call for the assessment of the reproductive toxicity of locally used pesticides.

## 1. Introduction

Infertility is a major health concern across the world. It is defined as no conception after at least 12 months of regular intercourse without contraceptive (1). About 80 million of couples (10%-15%) worldwide are victims of different types of infertility (2). About 20%-40% of sub-Saharan Africa's populations suffer from infertility (3). In Cameroon, the prevalence of infertility remains poorly known (4). Scientific evidence shows that the male factors are involved in 50%-60% of overall infertility; however, they are solely incriminated in case of only 20% of couples (1). Infertility has long been considered as a female-derived problem in Africa; hence, very little effort has been made to identify the male responsibility in infertile couples. In Cameroon, this domain remains unexplored due to cultural, ethical, and religious barriers. A study conducted at the Yaoundé Central Hospital revealed that men are responsible in about 20% of couples attending urology services for infertility problem (5).

Although 50% of cases are idiopathic, many factors, including anatomical, physiological, genetic, and environmental, have been associated with male infertility (6). For instance, the use of pesticides to protect plants from devastators has critically increased in the recent years, even though they are considered as the major environmental factor affecting the male reproductive tract. These pesticides mimic internal hormones leading to endocrine disorders associated with oxidative stress. Abell and colleagues (7) reported about 6% decrease in sperm parameters among farmers and inhabitants of farming areas. Although sperm abnormalities are generally considered as inaccurate predictors of fertility, semen analysis (SA) is however used as a screening test to identify the male factor involvement in infertility (2). Combined to the male reproductive history, SA is the primary screening for male fertility potential according to the American Society for Reproductive Medicine and the American Urological Association guidelines (8). About 30%-50% cases of male infertility worldwide are related to poor sperm quality (7). In Cameroon, Nana and collaborators reported that sperm damage accounted for infertility in 41.7% of infertile men in the urology services of the Yaoundé Central Hospital (5).

Considering the intensive agricultural practices in the West Region of Cameroon, the prevalence of sperm damage-related male infertility in the Dschang Health District was evaluated in this study. Based on the deleterious effects of pesticides on spermatogenesis, we hypothesized that the poor sperm quality could be the major factor associated with male infertility in the Dschang Health District.

This work was therefore undertaken to assess the sperm profile and related sociodemographic factors of infertile men attending some urology services of health centers in the Dschang Health District.

## 2. Materials and Methods

### Study design and data collection

This descriptive retrospective study was conducted in the Dschang Health District. Only the well-equipped hospitals for SA were selected for the study. The results of SA of all patients who consulted for infertility from January 2008 to December 2017 were included, whereas files lacking important information, such as the year of consultation or data regarding semen quality and quantity, were excluded from the study.

From patients' files, the date of consultation, age, profession, and semen parameters were recorded.

To ensure equal qualitative (asthenozoospermia, teratozoospermia, and necrozoospermia) and quantitative (hypozoospermia, hyperzoospermia, azoospermia, oligozoospermia, normozoospermia, and polyzoospermia) semen analysis, all data (before and after 2010) were reinterpreted according to the guidelines of WHO on the examination and processing of human semen published in 2010 (9), regardless of the previous decision of the medical doctor.

### Data treatment

From the collected data, the frequency of consultations was calculated. Data were expressed as demographic features and state of spermogram described by the clinician's decision. For sociodemographic features, patients were grouped based on their age and profession, the respective proportions were calculated. Sperm parameters were grouped into qualitative and quantitative abnormalities as indicated above. The frequency of each abnormality was calculated.

### Ethical consideration

This study was performed in accordance with the ethical authorization delivered on July 12, 2017 by the Cameroon National Ethic Committee for Human Health Research (code: 2017/07/926/CE/CNERSH/SP) and all studied participants freely accepted to participate.

### Statistical analysis

Data were expressed as percentage using Epi Info 7 software.

## 3. Results

### Frequency of consultations

Male infertility is a relatively frequent pathology in our context. According to our results, a total of 379 patients were admitted for infertility from 2008 to 2017 in the Dschang Health District. In this group, abnormal spermogram was found in 83.9% of the patients. Contrary to the number of consultations which remarkably increased with years, the number of patients with abnormal spermogram showed a decreasing tendency (Table I).

### Age of patients

The correlation between age and fertility is well established (10). The average age of patients was 35 yr (SD = 9.64) with extremes of 20 and 68 yr and almost 69.66% of patients were less than 40 yr. As a general tendency, there was no linear relationship between the age and the state of the spermogram. Patients aged above 50 yr had the maximum percentage of abnormal spermogram (Table I).

### Profession of patients

Out of the 379 patients included in this study, 204 (53.83%) were workers. In this subpopulation, teachers (43; 21.08%) followed by technicians (37; 18.14%) and traders (36; 17.65%) were the most represented (Table I). Although drivers (21; 10.29%) and farmers (19; 9.31%) were the least represented categories, they surprisingly showed the highest percentage of abnormal spermogram, 90.47% and 94.74%, respectively (Table I).

The frequency of consultation increased with years and the percentage of abnormal spermogram showed a relatively stable tendency. The average year of patients included in the present study was 35 yr with extremes of 20 and 68 yr. About 69.66% of patients were aged less than 40 yr and patients aged above 50 yr had the highest percentage of abnormal spermogram.

### Semen abnormalities

**Figure 1:**
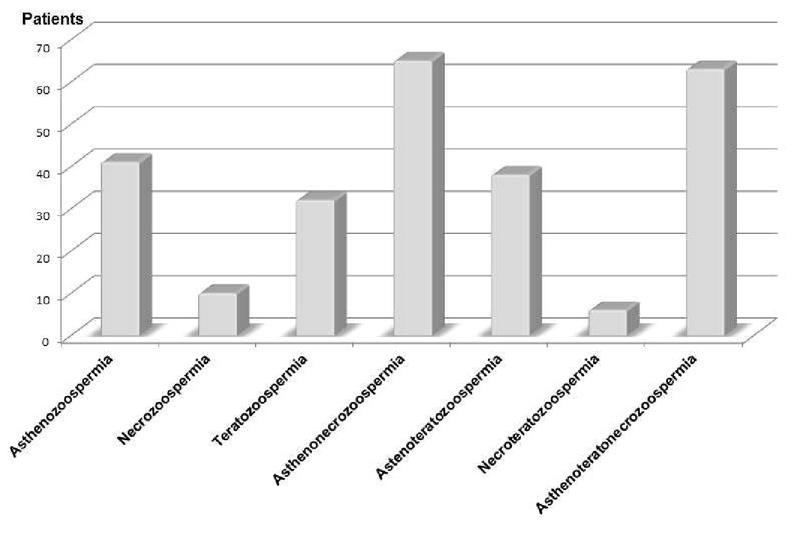
Patients grouping according to qualitative sperm abnormalities.

**Figure 2:**
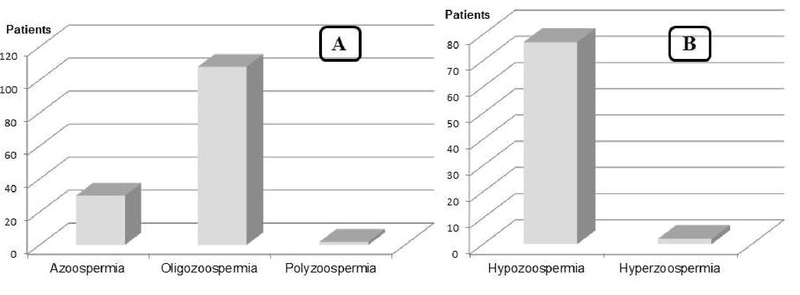
Patients grouping according to quantitative sperm abnormalities.

According to the guidelines of WHO on the examination and processing of human semen published in 2010, an abnormal spermogram was found in 318 (83.9%) patients. Asthenozoospermia (16.08%), asthenonecrozoospermia (25.49%). and asthenoteratonecrozoospermia (24.71%) were the most prevalent in patients having qualitative abnormalities (Figure 1), whereas hypozoospermia (95.06%) and oligozoospermia (77.14%) were the most recorded concerning the quantitative abnormalities (Figure 2 A, B).

According to our data, asthenozoospermia, asthenonecrozoospermia, and asthenoteratonecrozoospermia were the most prevalent qualitative sperm abnormalities. Oligozoospermia and hypozoospermia were the most prevalent quantitative sperm abnormalities in men consulting for infertility in the Dschang Health District from 2008 to 2017.

**Table 1:** Frequency of consultations by year and distribution of patients by age and profession


	**Normal spermogram**	**Abnormal spermogram**	**Total**
**Years**	**Frequency of consultations**		
**2008-2009**	1 (6.67)	14 (93.33)	15
**2010-2011**	7 (15.5)	38 (84.44)	45
**2012-2013**	8 (15.09)	45 (84.91)	53
**2014-2015**	16 (14.29)	96 (85.71)	112
**2016-2017**	29 (18.83)	125 (81.17)	154
**Total**	61 (16.09)	318 (83.91)	379
**Age range (yr)**	**Age of patients**		
**20-30**	25 (17.86)	115 (82.14)	140
**31-40**	19 (15.32)	105 (84.68)	124
**41-50**	11 (17.74)	51 (82.26)	62
**≥50**	0 (0.00)	19 (100.00)	19
**Other**	6 (17.65)	28 (82.35)	34
**Total**	61 (16.09)	318 (83.91)	379
	**Profession**		
**Teachers**	9 (20.93)	34 (79.07)	43
**Technicians**	4 (10.81)	33 (89.19)	37
**Traders**	4 (11.11)	32 (88.89)	36
**State agents**	7 (25.93)	20 (74.07)	27
**Students**	4 (19.05)	17 (80.95)	21
**Drivers**	1 (9.53)	20 (90.47)	21
**Farmers**	1 (5.26)	18 (94.74)	19
**Total**	30 (14.71)	174 (85.29)	204
Data presented as *n* (%)			

## 4. Discussion

Infertility is a prevalent problem concerning about 15% of couples during their reproductive age, and in half of this situation, a male factor is responsible (1). As few data are available about the prevalence of male infertility in Cameroon, this study was designed to evaluate the sperm profile and related sociodemographic factors of infertile men attending the urology services of health centers in Dschang Health District. The results of the present study showed that 83.9% of participants suffered from sperm damages. The majority of patients were aged under 40 yr and teachers were the most represented socioprofessional category. However, patients older than 50 yr and farmers had the highest percentages of abnormal spermogram.

The demand in urology services in the district was crescent from 2008 to 2017 (from 15 cases in 2008 to 154 in 2017). This tendency is confirmed by the literature which shows that there is an increasing adhesion of infertile couples to various infertility therapies provided by health centers across the world (11). In Cameroon and Africa, in general, this increasing demand could be explained by the fact that women are no longer solely blamed in childless couples (12). This attitude could also be motivated from the awareness of populations and the sociocultural aspects of infertility (13). The relationship between age and male fertility is well documented. The decline in male fertility potential with increasing age is a well-accepted concept and is consecutive to impairment of sperm parameters and genomic alterations (10). The mean age of the patients included in this study was 35 with extremes of 20 and 68. Similar data were found by Ahmed and colleagues (13) in Nigeria (34.0). Similar to the findings of Moussa and colleagues in Niamey, 69.66% of our patients were aged less than 40 yr (14). The high proportion of young patients in this study could be justified by the prenuptial check-up and the need for fatherhood.

Environmental factors are one of the determinants to male infertility. Thus, exposure to the inadequate socioprofessional environment is harmful to male fertility. Out of the 379 patients included in this study, the working status of 204 (53.83%) was found in the files. From this group, teachers (21.08%) were the most represented. This high prevalence could be associated with their knowledge and awareness on the involvement of both male and female factors in infertile couples. This result is similar to that of Sissoko and colleagues and Kaham and co-workers in Mali who reported that 28% and 30% of patients, respectively, consulting for infertility were civil servants (15, 16). However, our data are lower than those of Moussa and colleagues (14) in Niamey (60.93%). Although drivers (21; 10.29%) and farmers (19; 9.31%) were the least represented categories, they showed the highest percentage of abnormal spermogram, 90.47% and 94.74%, respectively. Infertility is still considered as a taboo in those categories where the common belief points women as the problem. The high prevalence of sperm damage in farmers could be a direct consequence of the exposure to pesticides (17). The toxic impact of pesticides is generally due to the release of free radicals in biological systems and the resulting oxidative stress is responsible for necrosis in surrounding cells, such as Leydig and Sertoli cells in the testis (18). Concerning the drivers, the prolonged exposure to hydrocarbon combined with high temperatures could explain present results. Many studies have reported the detrimental effects of high temperature and heat stress on male fertility (19). Normally, the scrotal temperature must be 2.2 °C lower than the abdominal temperature to provide physiological conditions for spermatogenesis. Hence, an increase in scrotal temperature leads to either impaired sperm production or abnormal sperm morphology (20).

In the present study, asthenozoospermia, oligozoospermia, and hypozoospermia were the most prevalent factors associated with male infertility. According to the WHO (2010), asthenozoospermia is the decrease in sperm mobility less than 30% of total sperm. Oligozoospermia and hypozoospermia refer to a reduced number of sperms and semen volume, respectively (1). These features are generally a consequence of poor spermatogenesis and are considered as infertility factors in male. Similar results were published by Masoumi and colleagues (1) who reported in a survey conducted on the main causes of infertility in patients referred to the infertility center in Fatemieh Hospital in Hamadan-Iran that, as semen disorder, asthenozoospermia was the most prevalent factor associated to male infertility. Instead, of oligozoospermia, Moussa and colleagues (14) found azoospermia as the most prevalent quantitative semen disorders among men consulting for infertility in the Radio-Isotopes Institute of Niamey. The poor spermogram condition observed in farmers could be related to their working conditions as they continue to use inadequate tools in their activities.

Male infertility remains a serious matter of concern in Africa, in general, and Cameroon, in particular. Till date, little effort has been made by authorities to address the situation. This study indicates that male infertility is a major health problem in the Dschang Health District with about 83.9% of patients consulting for infertility suffering from sperm damage. This situation highly expressed in farmers could be partially justified by the intensive use of pesticides in their activities. Therefore, there is a need for sensitization not only for the safe use of pesticides and other pollutants, but also for the reinforcement of the technical capacities of hospitals for better diagnosis and management of male infertility in Cameroon.

##  Conflict of Interest

Authors have no competing interests to declare.

## References

[1] Sz Masoumi, P Parsa, N Darvish, S Mokhtari, M Yavangi, G. Roshanaei

[2] Sikka Sureshc, Hellstrom Waynejg (2016). Current updates on laboratory techniques for the diagnosis of male reproductive failure. Asian Journal of Andrology.

[3] Emokpae Abiodunmathias, Uadia Patrickojeifo (2015). Male infertility in Nigeria: A neglected reproductive health issue requiring attention. Journal of Basic and Clinical Reproductive Sciences.

[4] E Belley Priso, Ct Nguefack, C Nguemgne, Tn Njamen, W Taila, E Banag

[5] Nana P. N., Wandji J. C., Fomulu J. N., Mbu R. E., Leke R. J. I., Woubinwou M. J. (2011). Aspects Psycho-Sociaux chez Patients Infertiles à la Maternite Principale de l'Hopital Central de Yaoundé, Cameroun. Clinics in Mother and Child Health.

[6] Ua Eze, Fe Okonof

[7] Abell A, Ernst E, Bonde Jens Peter (2000). Semen quality and sexual hormones in greenhouse workers. Scandinavian Journal of Work, Environment & Health.

[8] (2015). Diagnostic evaluation of the infertile male: a committee opinion. Fertility and Sterility.

[9] Ford W. C. L. (2010). Comments on the release of the 5th edition of the WHO Laboratory Manual for the Examination and Processing of Human Semen. Asian Journal of Andrology.

[10] Belloc Stephanie, Hazout Andre, Zini Armand, Merviel Philippe, Cabry Rosalie, Chahine Hikmat, Copin Henri, Benkhalifa Moncef (2014). How to overcome male infertility after 40: Influence of paternal age on fertility. Maturitas.

[11] Leridon Henri (1991). Stérilité et hypofertilité: du silence à l'impatience?. Population (French Edition).

[12] Ikechebelu Ji, Adinma Jib, Orie Ef, Ikegwuonu So (2003). High prevalence of male infertility in southeastern Nigeria. Journal of Obstetrics and Gynaecology.

[13] Kirakoya Brahima, Barnabé Zango, Abdoul Karim Paré, Aristide Kaboré Fasnéwendé, Clotaire Yaméogo, Amélie Nikièma (2015). Epidemiological and Clinical Profile of Male Hypofertility in Consultation at the Urology-Andrology of Yalgado Ouedraogo Teaching Hospital (Burkina Faso). Advances in Sexual Medicine.

[14] Moussa D., Soumana A., Amadou S.M., Soli I., Tahirou I., Ali A. (2016). Profil hormonal chez l’homme en cas d’infertilité au laboratoire de radio immunologie de l’institut des radioisotopes de Niamey. African Journal of Urology.

[15] Sb Sissoko

[16] P. Kaham

[17] Ould Hamouda S., Perrin J., Achard V., Courbière B., Grillo J.-M., Sari-Minodier I. (2016). Association entre anomalies spermatiques et environnement professionnel chez les hommes consultant pour infertilité de couple. Journal de Gynécologie Obstétrique et Biologie de la Reproduction.

[18] Hafez Essam M, Issa Sahar Y , Mazroua Maha K Ai, Ibrahim Karem T , Rahman Safaa M Abdel  (2016). The Neonicotinoid Insecticide Imidacloprid: A Male Reproductive System Toxicity Inducer-Human and Experimental Study. Toxicology: Open Access.

[19] Hamerezaee Masoud, Dehghan Somayeh F., Golbabaei Farideh, Fathi Asad, Barzegar Loghman, Heidarnejad Naseh (2018). Assessment of Semen Quality among Workers Exposed to Heat Stress: A Cross-Sectional Study in a Steel Industry. Safety and Health at Work.

[20] Hamilton Thais Rose Dos Santos, Mendes Camilla Mota, Castro Letícia Signori De, Assis Patrícia Monken De, Siqueira Adriano Felipe Perez, Delgado Juliana De Carvalho, Goissis Marcelo Demarchi, Muiño-Blanco Teresa, Cebrián-Pérez José Álvaro, Nichi Marcílio, Visintin José Antonio, Assumpção Mayra Elena Ortiz D’Ávila (2016). Evaluation of Lasting Effects of Heat Stress on Sperm Profile and Oxidative Status of Ram Semen and Epididymal Sperm. Oxidative Medicine and Cellular Longevity.

